# Transcranial Magnetic Stimulation to the Middle Frontal Gyrus During Attention Modes Induced Dynamic Module Reconfiguration in Brain Networks

**DOI:** 10.3389/fninf.2019.00022

**Published:** 2019-04-03

**Authors:** Penghui Song, Hua Lin, Chunyan Liu, Yuanling Jiang, Yicong Lin, Qing Xue, Peng Xu, Yuping Wang

**Affiliations:** ^1^Department of Neurology, Xuanwu Hospital, Capital Medical University, Beijing, China; ^2^Key Laboratory for Neuroinformation of Ministry of Education, School of Life Science and Technology, University of Electronic Science and Technology of China, Chengdu, China; ^3^Center of Epilepsy, Beijing Institute for Brain Disorders, Capital Medical University, Beijing, China; ^4^Beijing Key Laboratory of Neuromodulation, Beijing, China

**Keywords:** middle frontal gyrus, TMS-EEG, attention, time-varying network, reorganization

## Abstract

The interaction between dorsal and ventral attention networks (VANs) is mediated by the middle frontal gyrus (MFG), which is functionally connected to both networks. However, the direct role of the MFG in selective and sustained attention remains controversial. In the current study, we used transcranial magnetic stimulation (TMS) and electroencephalography (EEG) to probe the connectivity dynamic changes of MFG-associated regions during different attention modes. The participants underwent visual, selective, and sustained attention tasks to observe TMS-induced network changes. Twenty healthy participants received single-pulse TMS over the left or right MFG during tasks, while synchronous EEG data was acquired. Behavioral results were recorded and time-varying brain network analyses were performed. We found that the MFG is involved in attention processing and that sustained attention was preferentially controlled by the right MFG. Moreover, compared with the right hemisphere, the left hemisphere was associated with selective attention tasks. Visual and selective attention tasks induced MFG-related changes in network nodes were within the left hemisphere; however, sustained attention induced changes in network nodes were in the bilateral posterior MFG. Our findings indicated that the MFG plays a crucial role in regulating attention networks. In particular, TMS-induced MFG alterations influenced key nodes of the time-varying brain network, leading to the reorganization of brain network modules.

## Introduction

Understanding the physiological mechanism of complex brain functions, such as attention, is a major challenge in neuroscience. Attention plays a crucial role in our ability to organize thoughts and actions into meaningful behaviors (Kim et al., 2016). Maintaining attention, including selective and sustained attention, is one of the most widely used abilities in humans. Chronic attention difficulties are characteristic of many neurodevelopmental disorders, such as autism spectrum disorder and attention deficit hyperactivity disorder (ADHD; Kooistra et al., 2010; Keehn et al., 2013). Attentional mechanisms are required to selectively enhance the most task-relevant information (Jia et al., 2017). Nonetheless, despite research indicating the importance of the middle frontal gyrus (MFG) for maintaining the integrity of attention networks (Gogulski et al., 2017), no study has systematically compared the role of the MFG in different attention modes (such as selective or sustained attention).

Functional magnetic resonance imaging (fMRI) has provided evidence that the MFG is active in block and event-related analyses of attention tasks, suggesting its importance in sustained attention/vigilance (Neale et al., 2015). Most brain network studies use fMRI-based analyses for functional connectivity because it has higher spatial resolution; however, the relatively slow temporal course of fMRI limits its ability to characterize network operation and observe dynamic processes. In addition, it is susceptible to artifacts produced from head movements (Rathee et al., 2017), and it utilizes either resting or task states of participants without external interfering stimuli. Therefore, fMRI is imperfect for studying top-down attention.

Transcranial magnetic stimulation (TMS) pulses can induce the synchronization of distant cortical areas, and thereby modulate information processing and alter functional connectivity patterns in specialized, interconnected cortical modules (Massimini et al., 2005). Therefore, TMS is a unique method for studying brain-behavior dynamics in humans (Pascual-Leone et al., 1999, 2000; Walsh and Cowey, 2000; Wu et al., 2016). To date, brain connectivity between different regions using electroencephalography (EEG) has shown causal communication mechanisms between distinct attention networks (Pang and Snead, 2016; Christoforou et al., 2017). TMS combined with EEG (TMS-EEG) will provide an important method to study brain networks.

New hardware developments, such as improved EEG amplifier technology and advanced data processing techniques, have removed the TMS-induced artifacts that had previously rendered concurrent TMS-EEG impossible (Rogasch and Fitzgerald, 2013). In addition, EEG analytical methods have developed from a directed transfer function (Kaminski and Blinowska, 1991) to an adapted, directed transfer function (ADTF; Wilke et al., 2007). This method can be used to measure connections between different brain regions at different frequencies in time (Zhang et al., 2017; Li et al., 2018). Hot spots or key nodes can be identified from active regions. These are the core elements of a whole network in a certain time epoch, which can dynamically change with time (Wang et al., 2017; Yan et al., 2018). Furthermore, modules can be identified as a group of nodes that are more strongly connected between each other than nodes in different modules within the network (Rathee et al., 2017). Subsequently, a single-pulse TMS (sTMS) alters neural activity in the stimulated area and modulates the excitability of interconnected distant sites (Siebner et al., 2001). Further, TMS-EEG can be applied to quantify this brain network connectivity (Thut and Miniussi, 2009).

This study aimed to directly test the contributions of MFG to different attention modes in healthy subjects and whether this contribution is asymmetrical relative to different modes. We hope that this research will contribute to a deeper understanding of time-varying brain connections and dynamic changes in key nodes in cortical areas related to the MFG.

## Materials and Methods

### Participants

Twenty healthy, right-handed individuals (10 males, mean age = 27.3 years, SD = 3.81) with normal or corrected-to-normal visual acuity were paid to participate in our experiment. All participants provided written informed consent for the study and publication. The study had the approval of the Xuanwu Hospital Ethics Committee and was in accordance with the Declaration of Helsinki.

### Attention Modes

**Figure 1:**
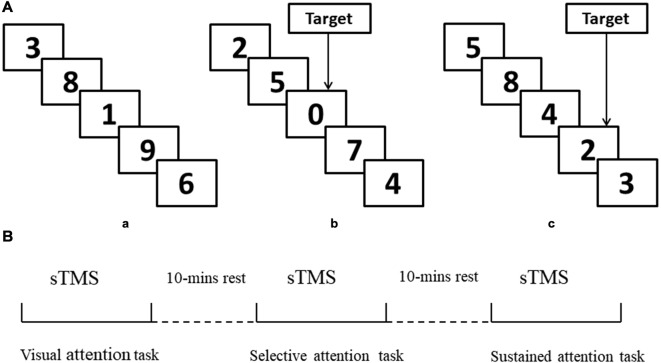
Schematic representation of experimental design. **(A,a)** Example of only visual attention task. **(b)** Example of target response in selective attention task block (number “0”). **(c)** Example of target response in sustained attention task block (triplets “8, 4, 2”). **(B)** Illustration of the concurrent transcranial magnetic stimulation (TMS)-electroencephalography (EEG) protocol and attention modes during sTMS. sTMS, single TMS.

We used three different attention modes in our experiment:

(1) Visual attention task. Participants were instructed to attend to the numbers that were presented between 0 and 9 randomly, with no choice component (Figure 1Aa).
(2) Selective attention task. Participants were instructed to attend to the numbers that were presented between 0 and 9 and respond whenever they saw a “0” (Figure 1b).
(3) Sustained attention task. Participants were instructed to attend to the numbers that were presented 1–9 and respond when they saw three consecutive odd or even numbers (“triplets”) in any sequence (e.g., 1, 3, 5 or 8, 4, 2; see Figure 1c).

All stimuli were controlled by a stimulus system (STIM, Neurosoft Labs Inc., Sterling, VA, USA) that presented numbers pseudo-randomly and with equal probability. The onset-to-onset interval and duration were 600 ms, without an inter-stimulus interval in all three conditions. All numbers were presented in white font on a black background. To ensure that the selective and sustained attention task blocks were matched for motor activation, both block types presented eight targets (“0” or odd/even triplets) appearing at a rate of four per 30 s. The selective and sustained attention conditions both included four blocks, with each block containing 200 numbers. There was a 1-min rest period between blocks without TMS stimulus.

### Neuronavigation

Participants’ heads were co-registered with their T1 MRI images using BrainSight™ frameless stereotaxic software (Rogue Research, Montreal, QC, Canada) to confirm the anatomical locus of stimulation. A Magstim Super-Rapid Stimulator (Magstim Co., Whitland, Dyfed, UK) was used to deliver the magnetic stimulation. TMS sessions corresponded to two targeted areas: (1) left MFG (center of BA 9); and (2) right MFG (center of BA 9).

### Measurements of Rest Motor Threshold

sTMS was applied with a figure-of-eight coil (70 mm diameter) connected to a monophasic Magstim stimulator (Magstim Company Ltd., London, UK). The stimulating coil was positioned tangentially to the skull with the coil handle pointing backward and laterally at 45° from the anterior-posterior axis. The left “motor hot spot” was determined as the site where the TMS consistently elicited the largest motor evoked potentials (MEPs) from the right first dorsal interosseous (FDI) muscle. This spot was marked on the scalp with a waterproof pen alongside the front edge of the TMS coil. The surface electromyography was recorded using disc-shaped Ag-AgCl electrodes that were placed in a tendon-belly arrangement. The resting motor threshold (RMT) was defined as the lowest stimulus intensity that elicited a minimum MEP amplitude of 50 μV in the completely relaxed FDI muscle in at least 5 out of 10 consecutive trials.

### EEG Data Acquisition

EEG data were acquired using a magnetic field-compatible EEG amplifier (Yunshen Ltd, Beijing, China) and cap (Greentek Ltd, Wuhan, China) with 32 TMS-compatible electrodes positioned according to the 10/20 system and digitized with a sample rate of 1,024 Hz. The CPz and nasal tip electrodes served as the reference and ground, respectively. During the entire experimental task, electrode impedances were maintained below 5 kΩ.

### Experimental Procedure

Participants were positioned on a semi-reclined chair with their forearms lying on armrests; care was taken to maintain a relaxed posture. Participants wore earplugs to avoid ambient and coil discharge noises. They were instructed to stay motionless without falling asleep. Each participant first completed the selective and sustained attention tasks without TMS to compare reaction times and correct response rates with responses during TMS application. We verified that the subjects remained alert by continuous EEG monitoring.

TMS was performed using a monophasic Magstim stimulator (Magstim Company Ltd, London, UK), which generates a maximum magnetic field of 1.5 T. sTMS was delivered through a figure-of-eight focal coil over the left or right MFG. The order of sTMS was randomized and there was a 30-min interval between each experiment (90% RMT). The sTMS interval was 4 s to avoid any TMS effect. Participants completed the three attention tasks during sTMS. The order of tasks was randomized, and there was a 10-min interval between tasks (Figure 1B). Left and right MFG were disturbed separately with a 30-min interval between experiments.

### EEG Data Analysis

#### Time-Varying Network Analysis

EEG data analysis was divided into pre-processing and time-varying network analyses. The time-varying network analysis required several segmentations to enable the construction of a reliable network to capture the brain architectures and networks. In this study, we used TMS disturbances as stimulus labels. For each labeled disturbance event, the time point corresponding to the peak of the label was set as time “0.” Then, data corresponding to 0.5 s before and 1 s after “0” were extracted (total segment length, 1.5 s). Next, to reduce the calculation load in the time-varying network analysis, segments were eight-rate down-sampled (Li et al., 2016), resulting in 32 Hz. ADTF was used to construct the time-varying networks and uncover the dynamic information processing during TMS disturbance (Wilke et al., 2007). We used a time-varying multivariate adaptive autoregressive model and ADTF to calculate the time-varying brain network (Zhang et al., 2017); this process is included in the Supplementary Material Appendix. The normalized total information outflow of the *j*th node is further estimated in Equation 1 as:

$${\text{Q}}_{j}^{2}(t)=\frac{\sum_{k=1}^{n}{\text{Q}}_{kj}^{2}(t)}{n-1},\text{for}\;k\neq j$$

where *n* is the total number of nodes.

When each node (*n*) has been calculated for each sample time point (*t*), a directional edge (*i* to *j*) can be displayed. From Equation 1, we can derive an outflow that denotes the time-varying of each node across different time points, as demonstrated in Figure 3. We defined the key node as the node with the highest degree of connectivity at various time points. The key node will change over time and at that sample time point, the edges quantity of this key node determines their connection strength.

**Figure 2:**
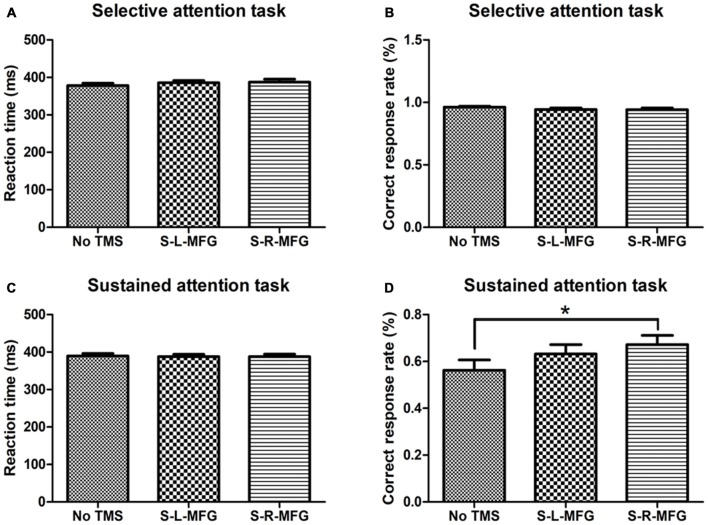
Reaction time and accuracy on each attention task. **(A)** Mean reaction time of correct responses in the selective attention task. **(B)** Correct response rate in the selective attention task. **(C)** Mean reaction time of correct responses in the sustained attention task. **(D)** Correct response rate in the sustained attention task. S, single TMS, L, left hemisphere, R, right hemisphere, MFG, middle frontal gyrus. *p < 0.05.

**Figure 3:**
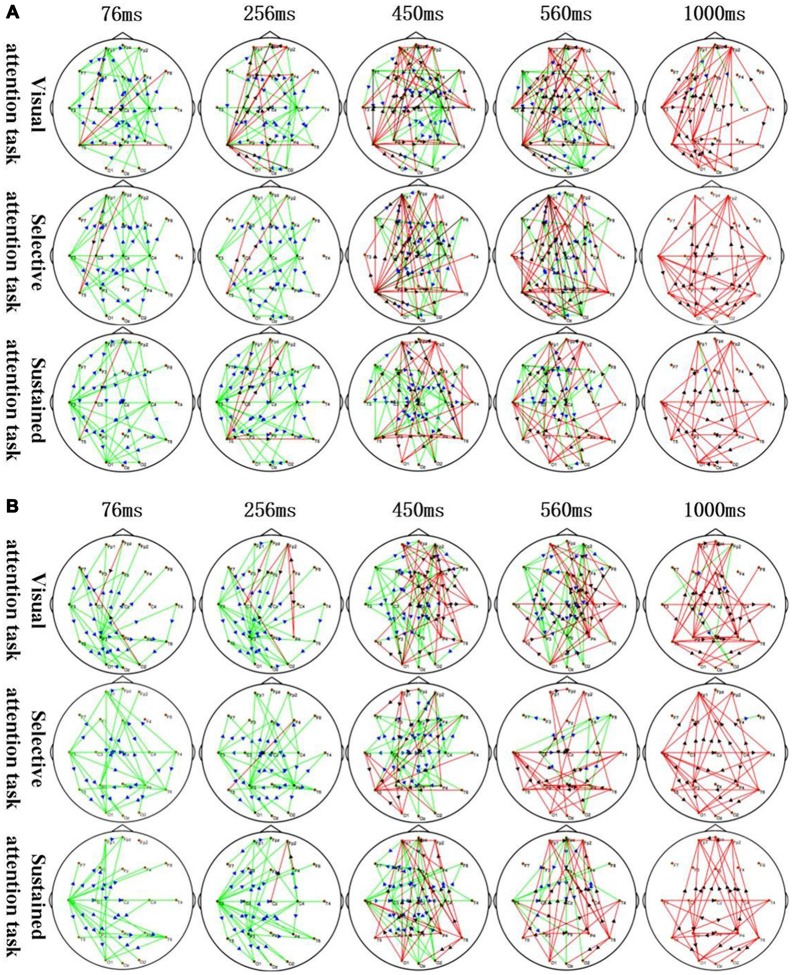
Application of sTMS to the left MFG **(A)** and right MFG **(B)** induced changes in the time-varying networks in different attention modes. Time: after single TMS. Red lines: enhanced connections; black arrows: the direction of information flow; green lines: weakened connections; blue arrows: the direction of information flow.

#### Behavioral Data Analyses

All results from the attention tasks were averaged across the 20 participants, and statistical analyses were used to identify differences in dynamic network patterns between attention modes.

*Post hoc* pairwise comparisons were used to compare the reaction times and correct response rates between attentional modes. Statistical significance was set at *p* < 0.05.

## Results

### Behavioral Results

We recorded the response time and accuracy of different attention tasks that were used to evaluate the contribution of the MFG to attention processing. There were no significant differences in response time or accuracy in the selective attention task during sTMS in the right or left MFG. Interestingly, participants showed an improvement in accuracy in the sustained attention task when sTMS was applied to the right MFG (*p* < 0.05; Figure 2).

### Dynamic Network Patterns

The corresponding MFG time-varying network patterns of the different attention modes are shown in Figure 3. Specifically, application of sTMS to the left MFG induced changes in the time-varying networks in different attention modes. The left temporal and right central area connection was initially weakened (76–450 ms) but was followed by an enhanced bilateral temporal connection (450–1,000 ms). The left MFG induced a longer inhibition of the left temporal region in the sustained attention task, as compared to the other attention modes (Figure 3A). Additionally, application of sTMS to the right MFG induced time-varying network alterations in different attention modes. The connection between the left temporal and parietal lobes was initially weakened (76–256 ms) but was followed by an enhanced bilateral temporal connection (450–1,000 ms). MFG-induced inhibition of the left temporal connection was observed in the sustained attention task at 560 ms. This inhibition was observed at 450 ms in the other tasks (Figure 3B).

The time-varying network patterns from different attention modes are shown in Figure 3. These data reveal key network nodes located in different brain regions. Moreover, local brain regions close to the attention zones are activated at the differential time-points.

**Figure 4:**
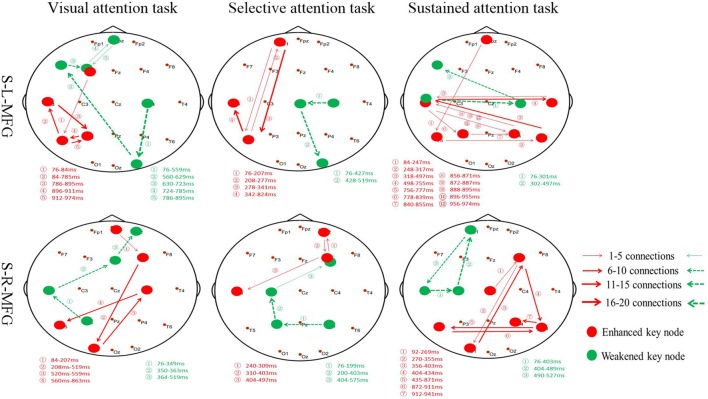
Key nodes were altered during different attention modes. Red digits: conversion time of enhanced key nodes. Green digits: conversion time of weakened key nodes. Red lines and arrows: the direction of enhanced key nodes information flow. Green lines and arrows: the direction of weakened key nodes information flow. S, single TMS, L, left hemisphere, R, right hemisphere, MFG, middle frontal gyrus.

Furthermore, the association between the MFG and key nodes were altered during different attention modes (Figure 4). For example, when sTMS was applied to the left MFG during the visual attention task, key nodes in the left frontal region to the left posterior region were enhanced, and key nodes from the right posterior region to the left frontal region were weakened. When sTMS was applied to the left MFG during the selective attention task, key nodes from the left frontal region to the left posterior region were enhanced and were weakened in the right posterior region. Enhanced key nodes from the left and right posterior region and weakened key nodes in the left and right frontal region were observed when sTMS was applied to the left MFG during the sustained attention task. Following sTMS application to the right MFG during the visual attention task, the key nodes from the right frontal region to the left posterior region were enhanced, and the key nodes from the left posterior region to the right frontal region were weakened. The selective attention task revealed a change in the enhanced key nodes from the right frontal region to left posterior region and weakened key nodes changed from the right posterior region to the right frontal region. The sustained attention task revealed alterations in the enhanced key nodes from the right frontal region to the bilateral posterior region, and weakened key nodes changed from the left frontal region.

Our analyses revealed similar results in the left and right MFG in both selective and sustained attention modes; however, the visual and selective attention tasks revealed a hemispheric asymmetry, with key nodes associated with MFG in the left hemisphere. The sustained attention task revealed bilateral key nodes and increased connections between hemispheres.

## Discussion

Previous studies have noted the importance of the prefrontal cortex and frontal-parietal network in attention and our results have shown that the MFG makes a significant contribution to attention processing. Furthermore, we found that sTMS application in the right MFG can improve sustained attention. Interestingly, MFG-associated visual and selective attentional network key nodes were altered in the left hemisphere from the frontal region to posterior regions; however, sustained attention key nodes showed bilateral information exchange with right or left sTMS application.

The prefrontal lobe has been linked to attention in humans; however, its mechanism and role have not been fully elucidated. The earliest therapeutic use of repetitive TMS (rTMS) for ADHD led to an improvement in clinical global depression and ADHD-IV scales (Weaver et al., 2012). Similarly, our results indicate that sTMS application to the right MFG can have a positive effect on sustained attention. The left hemisphere is more associated with selective attention, and our results indicate bilateral MFG activation in sustained attention tasks. Further, disturbance of the right MFG may activate the right hemisphere and facilitate network connections with other regions to improve sustained attention ability. These results indicate a possible therapeutic potential for sTMS in the right MFG in individuals with sustained attention deficits.

Selective and sustained attention are primarily controlled by the dorsal attention network (DAN) and the ventral attention network (VAN; Corbetta and Shulman, 2002). There is a hemispheric asymmetry between attention networks, which results in the functional lateralization of the MFG (Corbetta and Shulman, 2011; Koch et al., 2011; Thiebaut et al., 2011; Neale et al., 2015). Lesion-based studies in ADHD have indicated that unbalanced interhemispheric interactions between the bilateral MFG account for the hemispheric specialization of attention function (Epstein et al., 2009; Nagashima et al., 2014). Current data indicate that the functional asymmetry of MFG is linked with different brain networks. This is supported by our data showing asymmetric connectivity of the MFG between different attention modes.

sTMS application to the MFG induced changes to time-varying networks in different attention modes, which included enhanced and weakened connections. The visual and selective attention task revealed alterations in the location of enhanced connections from the frontal region to the posterior region in the left hemisphere and weakened connections from the posterior region to the front brain. In contrast, the sustained attention task revealed changes to enhanced connections bilaterally in the posterior region and altered weakened connections in the frontal region. This indicates that the MFG has different roles in different attention modes, and right MFG has its most important role in sustained attention processing (Caruana et al., 2015; Han et al., 2018). Previous data have shown that TMS affects performance when applied to either hemisphere (Duecker et al., 2013; Platz et al., 2016); however, we found a strong right MFG effect. This indicates that this frontal region may have a spatially biased functional role.

Previous studies of brain structure analysis, based on functional connectivity patterns, have shown modular organization. These are classified into four modules that are associated with different functions: occipital (perception), central and sensorimotor (action), and frontoparietal (executive functions) modules; and the default mode network (spontaneous cognition). This indicates that there is a well-defined network organization in the brain at rest and during task performance (Laird et al., 2005; Crossley et al., 2013). The present study revealed that reorganization of brain network modules might contribute to attention processing. Furthermore, there are differences in network topology between different attention modes. We have demonstrated that the left hemisphere plays a leading role in visual and selective attention processes (Fink et al., 1997; Yamaoka and Michimata, 2015; Sweeti et al., 2018).

The time-varying network in this study highlighted that the MFG plays an important role in dynamic network changes that are involved in attentional processing and may have a regulatory function in attention processing, particularly the right MFG in sustained attention. Studies have reported that the right posterior parietal cortex has stronger anatomical connections with the ipsilateral MFG than the left posterior parietal cortex (Wu et al., 2016). In the current study, we discovered that the right hemisphere preferentially mediates sustained attention, due to unbalanced interactions between the bilateral frontoparietal networks. Correct response rate can be improved by stimulating the right MFG during sustained attention tasks and increasing the interhemispheric parietal network connections. These asymmetric connections were associated with behavioral performances.

This study has a few limitations, some of which may merit future investigation. First, the brain regions to which sTMS was applied were relatively limited and only located in the prefrontal cortex. Next, although we used three attention modes in the present study, there are many attention-related tasks that can be used to assess network changes. Finally, the sample size of this study was small. Future studies should address these limitations.

## Conclusions

We sought to assess the role of the MFG in different attention modes by using sTMS to induce dynamic changes to brain networks. We have confirmed that the MFG is involved in attention processing, and our findings suggest that there is an asymmetry of sustained attention control towards the right MFG. Moreover, the left hemisphere is more involved in selective attention tasks than the right hemisphere. Our principal findings demonstrate that during visual and selective attention, MFG-related networks were situated in the left hemisphere, whereas sustained attention led to a greater activation of key nodes in the bilateral posterior region of the brain. These findings suggest that sTMS-induced MFG disturbances can cause key nodes in brain networks altered and reorganized.

## Ethics Statement

All participants provided written informed consent for the study and publication. The study had the approval of the Xuanwu Hospital Ethics Committee and was in accordance with the Declaration of Helsinki.

## Data Availability

All datasets generated for this study are included in the manuscript and/or the Supplementary Files.

## Author Contributions

YW conceived and designed the study. PS acquired the data and drafted the manuscript. HL, CL, YL, YJ, QX and PX contributed to data analysis and interpretation.

## Conflict of Interest Statement

The authors declare that the research was conducted in the absence of any commercial or financial relationships that could be construed as a potential conflict of interest.

## References

[B1] Caruana N. Brock J. Woolgar A. (2015). A frontotemporoparietal network common to initiating and responding to joint attention bids. Neuroimage 108, 34–46. 10.1016/j.neuroimage.2014.12.041 25534111

[B2] Christoforou C. Papadopoulos T. C. Constantinidou F. Theodorou M. (2017). Your brain on the movies: a computational approach for predicting box-office performance from viewer’s brain responses to movie trailers. Front. Neuroinform. 11:72. 10.3389/fninf.2017.00072 29311885 PMC5742097

[B3] Corbetta M. Shulman G. L. (2002). Control of goal-directed and stimulus-driven attention in the brain. Nat. Rev. Neurosci. 3, 201–215. 10.1038/nrn755 11994752

[B4] Corbetta M. Shulman G. L. (2011). Spatial neglect and attention networks. Annu. Rev. Neurosci. 34, 569–599. 10.1146/annurev-neuro-061010-113731 21692662 PMC3790661

[B5] Crossley N. A. Mechelli A. Vertes P. E. Winton-Brown T. T. Patel A. X. Ginestet C. E.. (2013). Cognitive relevance of the community structure of the human brain functional coactivation network. Proc. Natl. Acad. Sci. U S A 110, 11583–11588. 10.1073/pnas.1220826110 23798414 PMC3710853

[B6] Duecker F. Formisano E. Sack A. T. (2013). Hemispheric differences in the voluntary control of spatial attention: direct evidence for a right-hemispheric dominance within frontal cortex. J. Cogn. Neurosci. 25, 1332–1342. 10.1162/jocn_a_00402 23574586

[B7] Epstein J. N. DelBello M. P. Adler C. M. Altaye M. Kramer M. Mills N. P.. (2009). Differential patterns of brain activation over time in adolescents with and without attention deficit hyperactivity disorder (ADHD) during performance of a sustained attention task. Neuropediatrics 40, 1–5. 10.1055/s-0029-1220686 19639521 PMC2768528

[B8] Fink G. R. Halligan P. W. Marshall J. C. Frith C. D. Frackowiak R. S. Dolan R. J. (1997). Neural mechanisms involved in the processing of global and local aspects of hierarchically organized visual stimuli. Brain 120, 1779–1791. 10.1093/brain/120.10.1779 9365370

[B9] Gogulski J. Zetter R. Nyrhinen M. Pertovaara A. Carlson S. (2017). Neural substrate for metacognitive accuracy of tactile working memory. Cereb. Cortex 27, 5343–5352. 10.1093/cercor/bhx219 28968804

[B10] Han Q. Zhang Y. Liu D. Wang Y. Feng Y. Yin X.. (2018). Disrupted local neural activity and functional connectivity in subjective tinnitus patients: evidence from resting-state fMRI study. Neuroradiology 60, 1193–1201. 10.1007/s00234-018-2087-0 30159629

[B11] Jia J. Liu L. Fang F. Luo H. (2017). Sequential sampling of visual objects during sustained attention. PLoS Biol. 15:e2001903. 10.1371/journal.pbio.2001903 28658261 PMC5489144

[B12] Kaminski M. J. Blinowska K. J. (1991). A new method of the description of the information flow in the brain structures. Biol. Cybern. 65, 203–210. 10.1007/bf00198091 1912013

[B13] Keehn B. Muller R. A. Townsend J. (2013). Atypical attentional networks and the emergence of autism. Neurosci. Biobehav. Rev. 37, 164–183. 10.1016/j.neubiorev.2012.11.014 23206665 PMC3563720

[B14] Kim H. Ährlund-Richter S. Wang X. Deisseroth K. Carlén M. (2016). Prefrontal parvalbumin neurons in control of attention. Cell 164, 208–218. 10.1016/j.cell.2015.11.038 26771492 PMC4715187

[B15] Koch G. Cercignani M. Bonni S. Giacobbe V. Bucchi G. Versace V.. (2011). Asymmetry of parietal interhemispheric connections in humans. J. Neurosci. 31, 8967–8975. 10.1523/jneurosci.6567-10.2011 21677180 PMC6622945

[B16] Kooistra L. Crawford S. Gibbard B. Ramage B. Kaplan B. J. (2010). Differentiating attention deficits in children with fetal alcohol spectrum disorder or attention-deficit-hyperactivity disorder. Dev. Med. Child Neurol. 52, 205–211. 10.1111/j.1469-8749.2009.03352.x 19549201

[B17] Laird A. R. Lancaster J. L. Fox P. T. (2005). BrainMap: the social evolution of a human brain mapping database. Neuroinformatics 3, 65–78. 10.1385/ni:3:1:065 15897617

[B18] Li F. Chen B. Li H. Zhang T. Wang F. Jiang Y.. (2016). The time-varying networks in P300: a task-evoked EEG study. IEEE Trans. Neural Syst. Rehabil. Eng. 24, 725–733. 10.1109/TNSRE.2016.2523678 26849870

[B19] Li F. Peng W. Jiang Y. Song L. Liao Y. Yi C.. (2018). The dynamic brain networks of motor imagery: time-varying causality analysis of scalp EEG. Int. J. Neural Syst. 29:1850016. 10.1142/S0129065718500168 29793372

[B20] Massimini M. Ferrarelli F. Huber R. Esser S. K. Singh H. Tononi G. (2005). Breakdown of cortical effective connectivity during sleep. Science 309, 2228–2232. 10.1126/science.1117256 16195466

[B21] Nagashima M. Monden Y. Dan I. Dan H. Tsuzuki D. Mizutani T.. (2014). Acute neuropharmacological effects of atomoxetine on inhibitory control in ADHD children: a fNIRS study. Neuroimage Clin. 6, 192–201. 10.1016/j.nicl.2014.09.001 25379431 PMC4215398

[B22] Neale C. Johnston P. Hughes M. Scholey A. (2015). Functional activation during the rapid visual information processing task in a middle aged cohort: an fMRI study. PLoS One 10:e0138994. 10.1371/journal.pone.0138994 26488289 PMC4619344

[B24] Pang E. W. Snead I. O. (2016). From structure to circuits: the contribution of MEG connectivity studies to functional neurosurgery. Front. Neuroanat. 10:67. 10.3389/fnana.2016.00067 27445705 PMC4914570

[B25] Pascual-Leone A. Bartres-Faz D. Keenan J. P. (1999). Transcranial magnetic stimulation: studying the brain-behaviour relationship by induction of ’virtual lesions’. Philos. Trans. R. Soc. Lond., B, Biol. Sci. 354, 1229–1238. 10.1098/rstb.1999.0476 10466148 PMC1692644

[B26] Pascual-Leone A. Walsh V. Rothwell J. (2000). Transcranial magnetic stimulation in cognitive neuroscience–virtual lesion, chronometry and functional connectivity. Curr. Opin. Neurobiol. 10, 232–237. 10.1016/s0959-4388(00)00081-7 10753803

[B27] Platz T. Schüttauf J. Aschenbach J. Mengdehl C. Lotze M. (2016). Effects of inhibitory theta burst TMS to different brain sites involved in visuospatial attention - a combined neuronavigated cTBS and behavioural study. Restor. Neurol. Neurosci. 34, 271–285. 10.3233/rnn-150582 26923615

[B28] Rathee D. Cecotti H. Prasad G. (2017). “Propofol-induced sedation diminishes the strength of frontal-parietal-occipital EEG network,” in 2017 39th Annual International Conference of the IEEE Engineering in Medicine and Biology Society (EMBC) (Seogwipo South Korea: IEEE), 4463–4466. 10.1109/EMBC.2017.8037847 29060888

[B29] Rogasch N. C. Fitzgerald P. B. (2013). Assessing cortical network properties using TMS-EEG. Hum. Brain Mapp. 34, 1652–1669. 10.1002/hbm.22016 22378543 PMC6870446

[B30] Siebner H. Peller M. Bartenstein P. Willoch F. Rossmeier C. Schwaiger M.. (2001). Activation of frontal premotor areas during suprathreshold transcranial magnetic stimulation of the left primary sensorimotor cortex: a glucose metabolic PET study. Hum. Brain Mapp. 12, 157–167. 10.1002/1097-0193(200103)12:3<157::aid-hbm1012>3.0.co;2-v 11170307 PMC6871986

[B31] Sweeti Joshi D. Panigrahi B. K. Anand S. Santhosh J. (2018). Study of target and non-target interplay in spatial attention task. J. Med. Eng. Technol. 42, 113–120. 10.1080/03091902.2018.1433244 29448856

[B32] Thiebaut D. S. M. Dell’Acqua F. Forkel S. J. Simmons A. Vergani F. Murphy D. G.. (2011). A lateralized brain network for visuospatial attention. Nat. Neurosci. 14, 1245–1246. 10.1038/nn.2905 21926985

[B33] Thut G. Miniussi C. (2009). New insights into rhythmic brain activity from TMS-EEG studies. Trends Cogn. Sci. 13, 182–189. 10.1016/j.tics.2009.01.004 19286414

[B34] Walsh V. Cowey A. (2000). Transcranial magnetic stimulation and cognitive neuroscience. Nat. Rev. Neurosci. 1, 73–79. 10.1038/35036239 11252771

[B100] Wang L. Wang W. Yan T. Song J. Yang W. Wang B.. (2017). Beta-Band Functional Connectivity Influences Audiovisual Integration in Older Age: An EEG Study. Front. Aging Neurosci. 9:239. 10.3389/fnagi.2017.00239 28824411 PMC5545595

[B35] Weaver L. Rostain A. L. Mace W. Akhtar U. Moss E. O’Reardon J. P. (2012). Transcranial magnetic stimulation (TMS) in the treatment of attention-deficit/hyperactivity disorder in adolescents and young adults. J. ECT 28, 98–103. 10.1097/YCT.0b013e31824532c8 22551775

[B36] Wilke C. Ding L. He B. (2007). An adaptive directed transfer function approach for detecting dynamic causal interactions. Conf. Proc. IEEE Eng. Med. Biol. Soc. 2007, 4949–4952. 10.1109/iembs.2007.4353451 18003117

[B37] Wu Y. Wang J. Zhang Y. Zheng D. Zhang J. Rong M.. (2016). The neuroanatomical basis for posterior superior parietal lobule control lateralization of visuospatial attention. Front. Neuroanat. 10:32. 10.3389/fnana.2016.00032 27047351 PMC4805595

[B38] Yamaoka K. Michimata C. (2015). Spatial distribution of attention and inter-hemispheric competition. Cogn. Process. 16, 417–425. 10.1007/s10339-015-0734-5 26289477

[B101] Yan T. Wang W. Yang L. Chen K. Chen R. Han Y. (2018). Rich club disturbances of the human connectome from subjective cognitive decline to Alzheimer’s disease. Theranostics 8, 3237–3255. 10.7150/thno.23772 29930726 PMC6010989

[B39] Zhang L. Liang Y. Li F. Sun H. Peng W. Du P.. (2017). Time-varying networks of inter-ictal discharging reveal epileptogenic zone. Front. Comput. Neurosci. 11:77. 10.3389/fncom.2017.00077 28867999 PMC5563307

