# Supplementary material for: Transcranial Magnetic Stimulation to the Middle Frontal Gyrus During Attention Modes Induced Dynamic Module Reconfiguration in Brain Networks
Source: Front Neuroinform. 2019 Apr 3;13:22. doi: 10.3389/fninf.2019.00022 (PMC6456710; doi:10.3389/fninf.2019.00022)
Supplement: Supplementary file 1 [file Data_Sheet_1.PDF]

Appendix

Time-varying multivariate adaptive autoregressive (tv-MVAAR) Model

For each artifact-free segment, the tv-MVAAR model was defined as

$$X(t) = \sum_{i=1}^{p} A(i,t)X(t-1) + E(t) \tag{1}$$

where *X(t)* is the data vector of the EEG signal, *E(t)* is the multivariate independent white noise, *X(i,t)* is the matrix of the tv-MVAAR model coefficients that is estimated by the Kalman filter algorithm, and *p* is order of the model that is automatically determined by the Akaike Information Criterion (AIC), within the range of 2–20, as:

$$AIC(p) = \ln\left\lfloor \det(\chi) \right\rfloor + 2M^2 p / N \tag{2}$$

where *M* represents the number of electrodes, *p* represents the optimal order of the model, *N* represents the number of time points in each time series, and χ represents the corresponding covariance matrix.

Adaptive directed transfer function

Parameters *A(f,t)* and *H(f,t)* in the frequency domain are defined as follows;

$$A(f,t) = \sum_{k=0}^{p} A_k(t)e^{-j2\pi f \square tk} \tag{3}$$

$$A(f,t)X(f,t) = E(f,t) \tag{4}$$

$$X(f,t) = A^{-1}(f,t)E(f,t) = H(f,t)E(f,t) \tag{5}$$

where A_k denotes the matrix of the tv-MVAAR model coefficients, *X(f,t)* and *E(f,t)* are the Fourier transformations of *X(t)* and *X(t)* in the frequency domain, respectively.

Moreover, the normalized ADTF describing the directed flow from the *j*th to the *i*th node is defined in Equation (6), and the final integrated ADTF is defined in Equation (7) within the frequency band of interest as follows:

$$\gamma_{ij}^2(f,t) = \frac{\left|H_{ij}(f,t)\right|^2}{\sum_{m=1}^{n}\left|H_{im}(f,t)\right|^2} \tag{6}$$

$$Q_{ij}^2(t) = \frac{\sum_{k=f_1}^{f_2} \gamma_{ij}^2(k,t)}{f_2 - f_1} \tag{7}$$

The normalized total information outflow of the *j*th node is further estimated in Equation (8) as:

$$Q_j^2(t) = \frac{\sum_{k=1}^{n} Q_{kj}^2(t)}{n-1},\ for\ k \neq j \tag{8}$$

where n is the total number of nodes.
